# Two‐Stage Revision Arthroplasty for *Salmonella enteritidis* Periprosthetic Hip Infection

**DOI:** 10.1155/cro/6638707

**Published:** 2026-01-14

**Authors:** Stavros Lykos, Konstantinos Tsivelekas, Dimitrios Pallis, Spiridon Kamariotis, Georgios Macheras, Stamatios A. Papadakis

**Affiliations:** ^1^ Second Department of Orthopaedic Surgery, KAT General Hospital of Attica, Athens, Greece; ^2^ Department of Microbiology, KAT General Hospital of Attica, Athens, Greece; ^3^ 7th Department of Orthopaedic Surgery, Henry Dunant Hospital Center, Athens, Greece

## Abstract

Periprosthetic joint infections (PJIs) are severe complications following joint arthroplasty, commonly caused by Gram‐positive bacteria, such as *Staphylococcus aureus*. Infections due to Gram‐negative organisms like *Salmonella enteritidis* are exceedingly rare. In this study, we report a case of a 68‐year‐old male who developed a late‐onset PJI caused by *Salmonella enteritidis* 10 years after primary total hip arthroplasty (THA). A two‐stage revision arthroplasty with an interim antibiotic spacer was performed, followed by 6 weeks of intravenous antibiotic therapy. Three months postoperatively, the patient demonstrated full recovery with normal function. This case highlights the necessity of considering uncommon pathogens like *Salmonella enteritidis* in PJIs, even in patients without identifiable predisposing factors, and underscores the effectiveness of combined surgical and targeted antimicrobial therapy.

## 1. Introduction

Total hip replacement is a widely performed and effective intervention for advanced hip osteoarthritis, offering major improvements in mobility and quality of life. Although surgical and perioperative strategies have evolved, periprosthetic joint infections (PJIs) still pose a serious and potentially limb‐threatening complication [1]. PJIs are associated with increased morbidity, prolonged hospitalization, and substantial healthcare costs [2].

This case is notable for the unusually long latency (10 years after primary total hip arthroplasty [THA]), the absence of typical predisposing factors, and the need to individualize antibiotic therapy due to a documented fluoroquinolone (ciprofloxacin) allergy.

Currently, PJIs constitute the major indication for revision surgeries in more than 25% of them. Gram‐positive organisms, especially *Staphylococcus* species, are responsible for most PJIs, while Gram‐negative bacteria represent a much smaller proportion [3]. Gram‐negative organisms account for a smaller proportion of cases, with *Escherichia coli* and *Pseudomonas aeruginosa* being the most commonly identified among them [4]. Infections caused by *Salmonella* species are extremely rare, accounting for less than 0.3% of prosthetic joint infections in published series [4, 5].

The management of *Salmonella* spp. PJIs remains challenging due to its rarity. Thus, no clear consensus exists among authors regarding the treatment modality of choice, whether debridement‐antibiotics‐implant retention (DAIR), one‐stage, or two‐stage revision should be performed. Successful treatment with all modalities has been reported in the literature [4, 5, 6]. In this study, we present a rare case of chronic PJI caused by *Salmonella enteritidis* in a patient with no significant comorbidities. A two‐stage revision arthroplasty was performed, accompanied by selective intravenous antimicrobial therapy.

## 2. Case Presentation

A 68‐year‐old male presented to the outpatient clinic of our department complaining of progressively worsening pain in his right hip over the past 3 months. The patient observed mild swelling 2 weeks ago. An ipsilateral primary cementless THA had been performed 10 years prior due to hip osteoarthritis. The postoperative course was uneventful, and full functional recovery was achieved. His medical history was otherwise unremarkable, except for arterial hypertension managed with amlodipine. He denied any recent trauma, fever, chills, weight loss, or gastrointestinal symptoms such as diarrhea or abdominal pain. However, the patient reported a previous episode of *Salmonella* gastroenteritis diagnosed 3 years ago, which was successfully treated. There was no history of recent travel, consumption of undercooked poultry or eggs, or contact with sick individuals or animals. Notably, the patient has a documented allergy to ciprofloxacin.

### 2.1. Clinical Examination

During the clinical examination, the patient was afebrile with stable vital signs. Inspection of the right hip revealed mild swelling with erythema and warmth. A 2‐cm fluctuant, tender nodule was palpable over the lateral aspect of the hip, with purulent discharge upon percutaneous drainage. Leg length discrepancy was measured at 1 cm. At this time, the range of motion (ROM) was significantly reduced due to the pain, and the patient had a Harris Hip Score of 71/100. There were no signs of systemic infection.

### 2.2. Diagnosis

Laboratory tests revealed a white blood cell count of 10,500/*μ*L (normal range: 4000–11,000/*μ*L) with 80% neutrophils, elevated erythrocyte sedimentation rate (ESR) of 60 mm/h (normal range: 0–20 mm/h), and elevated C‐reactive protein (CRP) level of 2.1 mg/dL (normal range: < 0.3 mg/dL). Blood cultures were obtained and sent for microbiological analysis but yielded no growth.

**Figure 1:**
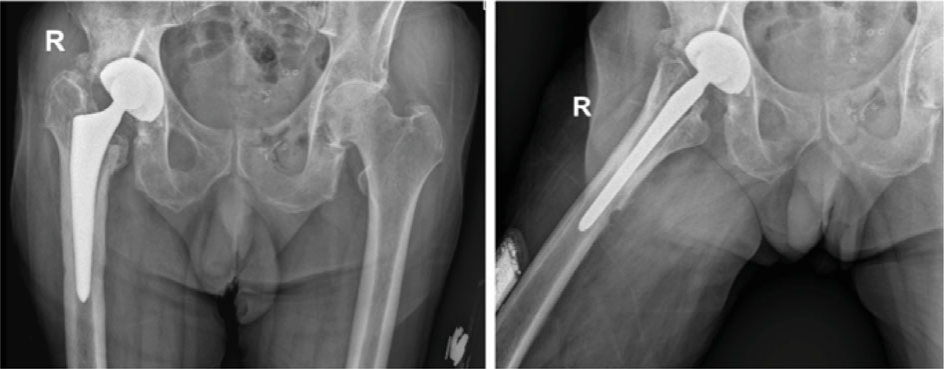
Anteroposterior and lateral x‐rays of the affected hip approximately 10 years after primary THA, prior to first‐stage revision. Arrows indicate periosteal reaction around the femoral stem and subtle signs of acetabular loosening.

Radiological assessment with anteroposterior and lateral x‐rays of the hip revealed a periosteal reaction around the femoral stem and signs suggestive of loosening of the acetabular component (Figure 1).

From a diagnostic standard standpoint, the case fulfilled contemporary PJI criteria as defined by the Musculoskeletal Infection Society [7] and endorsed by the Infectious Diseases Society of America, with one major criterion (presence of a sinus tract communicating with the prosthesis) and several *minor criteria* (elevated inflammatory markers, synovial leukocytosis with neutrophil predominance, and positive intraoperative cultures). Sonication of the explanted prosthesis and intraoperative calprotectin testing were employed as adjunctive methods that can improve sensitivity for low‐grade infections and support intraoperative decision‐making.

A CT‐guided aspiration of the hip joint was performed, yielding purulent fluid. Synovial fluid analysis showed a leukocyte count of 12,000 cells/*μ*L with 92% neutrophils. Synovial CRP was elevated to 3.4 mg/dL. Gram straining of the synovial fluid revealed Gram‐negative bacilli, and cultures were positive for *Salmonella* group, as detected by the fluctuant, tender nodule aspiration culture. Blood cultures were negative for any pathogen.

### 2.3. Treatment

**Figure 2:**
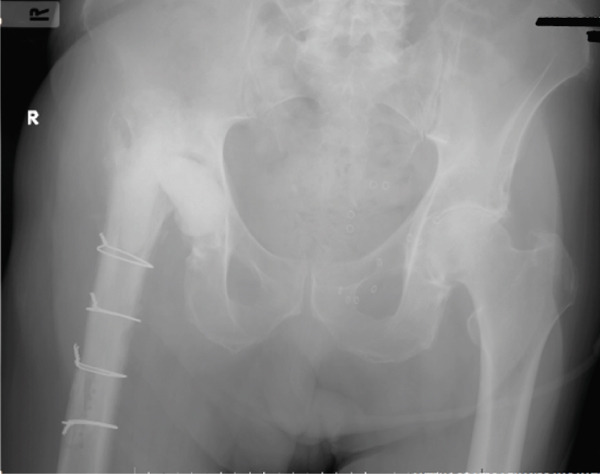
Postoperative anteroposterior pelvic x‐ray after the first stage of revision. Removal of both components was followed by internal fixation of the anterolateral femoral bone window with a stainless steel cerclage wire (1.5 mm) and implantation of a static gentamicin‐loaded PMMA cement spacer.

Given the diagnosis of a PJI caused by *Salmonella* spp., the Department of Infectious Diseases at our hospital recommended proceeding with a two‐stage revision arthroplasty within 48 h. During the first stage, both femoral and acetabular components were removed. The acetabular component was noticed to be loosened, while the cementless femoral stem required an anterolateral bone window to achieve removal of the stem. Six samples of tissue cultures and a bone culture were obtained. A thorough debridement of the infected and necrotic tissues was performed, followed by thorough washout with 9 L of saline. An antibiotic‐impregnated static custom‐made cement spacer (gentamicin‐loaded cement, 40 g polymethyl‐methacrylate [PMMA] with 0.5‐g gentamicin) was placed (Table 1). Postoperative radiographs after the first‐stage revision demonstrated removal of both components and implantation of a static antibiotic‐loaded cement spacer (Figure 2).

**Table 1:** Susceptibility testing of the presented case.

	**Res.**	**MIC**		**Res.**	**MIC**
Ampicillin/sulbactam	S	≤2	Gentamicin	R	≤1
Amikacin	R	≤2	Imipenem	S	≤0.25
Aztreonam	S	≤1	Levofloxacin	S	≤0.12
Cefepime	S	≤1	Piperacillin/tazobactam	S	≤4
Cefoxitin	S	≤4	Tigecycline	R	≤1
Ciprofloxacin (other)	S	≤0.25	Trimethoprim/sulfamethoxazole	S	≤20
Ceftazidime	S	≤1	Meropenem (other)	S	≤0.25
Fosfomycin (other)	R	≤16	Ceftriaxone (other)	S	≤1

Abbreviations: MIC, minimum inhibitory concentration (mcg/mL); R, resistant; S, susceptible.

### 2.4. Pathogen Identification

The explanted prosthesis was processed using a standardized sonication technique to enhance microbial recovery, as previously described in the literature [8]. Sterile Ringer′s solution was added to the container until the implant was nearly submerged. Following a brief vortexing phase, the implant underwent ultrasonic agitation (1‐min duration, 40 kHz, 0.22 W/cm^2^) using a BactoSonic bath (Bandelin GmbH). A final vortex ensured homogeneous distribution of any dislodged bacteria in the fluid, which was subsequently cultured on aerobic, anaerobic, and CO_2_‐enriched media. Microorganisms were identified using routine techniques. Optimal culture sensitivity and specificity were achieved if there were at least five colony‐forming units of the same organism on either plate. In our case, many colonies of the same bacteria were enumerated on blood and MacConkey agar. The intraoperative tissue and bone cultures showed growth of microorganisms. Cultures on blood agar showed growth of gray/white, nonhemolytic, nonswarming colonies that range from 2 to 3 mm in diameter after 24 h of incubation and colorless, transparent, lactose negative colonies between 2 and 3 mm in diameter after 24 h of incubation on MacConkey agar, which were Gram‐negative rods by Gram stain. Cultures on SS agar showed growth of black centered, flat, with transparent borders after 24 h of incubation. The identification and sensitivity of the *Salmonella* group were performed with the automated Vitek 2 system (Biomerieux, France). Identification of serotype was confirmed at the “Unit of Foodborne Diseases–National Reference Center Salmonella Shigella and Other Enteropathogens” of the National School of Public Health, Department of Public Health Policies, Laboratory for the Surveillance of Infectious Diseases (LSID), University of West Attica, Greece. The result of serotype was *Salmonella enteritidis* (9,12: g,m:‐). The sensitivity of the microbe was also checked by the reference center, and the antibiogram was sent to the laboratory in accordance to the European Committee on Antimicrobial Susceptibility Testing (EUCAST) (Table 1).

### 2.5. Antibiotic Therapy

The antimicrobial regimen was selected in close collaboration with the Department of Infectious Diseases, based on EUCAST susceptibility testing and the patient′s documented ciprofloxacin allergy. Postoperatively, intravenous coadministration of cefepime (2 g/24 h), ceftriaxone (1 g/24 h), and trimethoprim/sulfamethoxazole (700 mg/24 h) for 6 weeks was recommended by the Department of Infectious Diseases of our hospital to address the *Salmonella* PJI. Liver and renal functions, CRP, and ESR were monitored regularly, and no adverse reactions were observed. Normal levels of CRP were detected after 4 weeks of intravenous antibiotics. Following the 6‐week antibiotic course, the patient had a 2‐week antibiotic‐free interval to ensure eradication of the infection. Repeat blood cultures and joint aspiration were negative for bacterial growth. During this period, serial ESR and CRP levels were monitored and were observed to decline and stay within normal limits, respectively.

### 2.6. Treatment and Outcome

**Figure 3:**
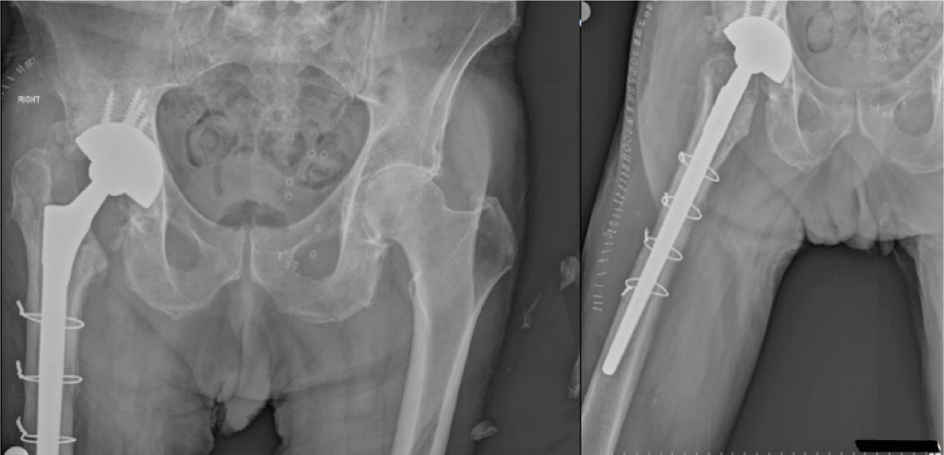
Postoperative anteroposterior and lateral hip x‐rays after the second stage (reimplantation) with a cementless grit‐blasted titanium femoral stem and a porous‐tantalum acetabular cup; radiographs obtained 6 weeks after reimplantation.

Nine weeks after the initial surgery, the patient underwent the second stage of revision arthroplasty. Before fascia incision, a calprotectin test of the synovial fluid was performed to ensure infection rule out. Calprotectin levels were measured at 13 mg/L, confirming the absence of active infection [9]. A cementless grit‐blasted biocompatible titanium alloy Wagner femoral stem and a porous‐tantalum acetabular cup were implanted (Figure 3).

The patient′s postoperative course was uneventful and received 48 h of intravenous prophylaxis with cefepime (2 g/day). Oral transition of the antibiotic therapy was consulted by the Department of Infectious Diseases and included ceftriaxone (1.5 g/24 h) and trimethoprim/sulfamethoxazole (800 + 160 ×2/24 h) for an additional 8 weeks. Physical therapy was initiated early to promote mobility and strength. The patient was discharged at the sixth postoperative day.

During the 3‐month follow‐up, the patient was ambulating independently without pain or signs of infection. Additionally, the HHS had improved to 83, reflecting satisfactory functional recovery. Laboratory tests revealed normal ESR and CRP levels. At the 12‐month follow‐up, the patient marked a HHS of 88 and inflammatory markers were at normal levels. A summary of the diagnostic, surgical, and therapeutic course of the patient is presented in Table 2.

**Table 2:** Timeline of key clinical events.

**Week**	**Event**
0	Onset of right hip pain and swelling
3	Diagnostic aspiration confirming *Salmonella enteritidis* infection
5	First‐stage revision with removal of components and spacer implantation
5–11	Six weeks of intravenous antibiotic therapy (cefepime, ceftriaxone, and TMP‐SMX)
9	Second‐stage reimplantation after negative cultures and calprotectin confirmation
12+	Follow‐up and complete functional recovery (HHS 88, normal ESR/CRP)

## 3. Discussion

*Salmonella enteritidis* is a Gram‐negative bacterium that most often causes gastrointestinal illness, typically via ingestion of contaminated food [5, 10]. Recently, a rise of *Salmonella* infection has been observed in several countries attributed to the increased global travel, with *Salmonella enteritidis* being the most commonly identified serotype [10, 11].

Antibiotic regimens should be individualized to susceptibilities and patient factors. Fluoroquinolones are often considered for Gram‐negative PJIs due to biofilm penetration, but contraindications (e.g., allergy) necessitate effective alternatives (e.g., third‐/fourth‐generation cephalosporins and/or TMP‐SMX) guided by microbiology and expert consultation. Although several centers currently favor a one‐stage revision approach for selected cases of PJI, our clinical experience has demonstrated more reliable outcomes with a two‐stage strategy in complex scenarios. In the present case, the presence of a purulent collection, combined with infection by a rare pathogen with high infective potential, led us to consider a two‐stage revision as the safer option for the patient. This approach was chosen with the aim of achieving complete eradication of the microbial agent, particularly given the limited data in the existing literature regarding the optimal management of *Salmonella enteritidis* PJIs.

In addition, the patient had a documented allergy to ciprofloxacin, which had initially been proposed by the infectious diseases specialist, adding uncertainty regarding the anticipated response to alternative antimicrobial regimens and further supporting a more cautious staged surgical approach.

### 3.1. Antibiotic Therapy and Biofilm Considerations

Both one‐stage and two‐stage strategies have been reported for Gram‐negative PJIs, including *Salmonella*. Two‐stage revision remains commonly favored in chronic infections or when implant loosening and biofilm burden are present, whereas carefully selected one‐stage procedures can achieve good outcomes when soft tissues are favorable, organisms are susceptible, and radical debridement is feasible.

### 3.2. Surgical Strategy: One‐Stage Versus Two‐Stage

Beyond meeting MSIS/IDSA criteria, adjunctive methods such as sonication and calprotectin can increase diagnostic yield and help stratify the likelihood of persistent infection at reimplantation.

### 3.3. Diagnostic Considerations

In *Salmonella* PJIs, hematogenous seeding from an enteric source is most frequently implicated, but subclinical bacteremia may account for delayed presentations in otherwise healthy hosts. The long latency in the present case underscores this possibility.

### 3.4. Epidemiology and Pathogenesis

Given its invasive characteristics, this bacterium can disseminate and affect extraintestinal sites in susceptible individuals [12]. Despite that the most common manifestation of salmonellosis is gastroenteritis, hematogenous spread and bacteremia can occur, particularly in patients with a compromised immune system or sickle cell disease [5, 10]. Risk factors for *Salmonella* PJIs include immunosuppression, malignancy, diabetes mellitus, hemoglobinopathies such as sickle cell disease, and chronic conditions like rheumatoid arthritis [13]. However, as in our report, individuals have been reported without significant predisposing factors, indicating the need for high clinical suspicion in atypical presentations [14].

While *Salmonella* infections are common globally, involvement of bones or joints, especially in the form of PJIs, remains exceptionally rare [15]. Gupta et al. observed that 0.2% of PJIs in a 44‐year retrospective study were due to *Salmonella* spp. [5]. Both septic arthritis and PJIs caused by *Salmonella* spp. are most commonly presented in patients with predisposing factors or recent associated medical reports of gastrointestinal complaints [4, 14, 16, 17]. As in our case, Jeroens et al. reported a *Salmonella enteritis* PJI without any previous signs of overt gastrointestinal symptoms [16]. The pathogenesis of *Salmonella* PJIs often involves hematogenous seeding from a primary infection site, usually the gastrointestinal tract [18]. However, our patient denied any recent episodes of gastroenteritis or exposure to common sources of *Salmonella* contamination. This suggests the possibility of subclinical bacteremia leading to colonization of the prosthetic joint, which emphasizes the need for vigilance in diagnosing atypical PJIs even in the absence of evident risk factors or primary infection sources [19].

Onset of *Salmonella* PJIs can be acute or chronic and there seems to be no outspoken trend in the literature. Most reports present delayed or chronic timeframes of PJIs [5, 6, 14, 20, 21]. However, Dojode et al. reported an acute PJI caused by *Salmonella enteritidis* 2 days after primary THA in a patient with regular dimethyl fumarate medication [10]. Moreover, Jeroens et al. described a *Salmonella* type E PJI 7 days after total hip replacement [16].

Treatment is complicated by the bacterium′s tendency to form protective biofilms on implants, which limits the efficacy of antibiotics [22]. There is no clear evidence in the literature for the optimal antibiotic therapy, yielding that the selected option should be individualized accordingly. Agents like fluoroquinolones are frequently preferred due to their favorable pharmacokinetics and tissue penetration, particularly within biofilms, while ciprofloxacin presents similar bioavailability and bone and soft tissue concentration when comparing intravenous and oral use [5, 23]. Widmer et al. reported the effectiveness of fluoroquinolones compared to trimethoprim–sulfamethoxazole (TMP‐SMX) in *Salmonella* PJIs [24]. Both treatment failures and successful outcomes in *Salmonella* PJIs have been reported after TMP‐SMX [5, 25]. Papavasileiou et al. observed the best results using ciprofloxacin and moxifloxacin for testing the antimicrobial resistance of *Salmonella enteritidis* [26]. Notably, several authors reported successful treatment without infection recurrence when using ciprofloxacin [5, 6, 10, 21]. Given the complexity of *Salmonella* PJI treatment, numerous other antibiotic options have been reported, including, as in our case, cephalosporins (third and fourth generation), clindamycin, ampicillin, vancomycin, TMP‐SMX, amoxicillin, and chloramphenicol resulting in infection treatment [4, 5, 14, 27].

Eradicating the infection usually involves combining surgical revision with targeted antimicrobial therapy [5]. Currently, several approaches have been reported including antibiotics, DAIR, and one or two‐stage revision surgeries [5, 6]. However, exclusive antibiotic therapy without any surgical debridement is usually insufficient. Gupta et al. observed failure of treatment in all patients where initial therapy consisted of aspiration and antimicrobial therapy only [5]. Retention or resection of the implants is currently dependent on the duration of symptoms, presence of implant loosening, tissue status, and bacterial susceptibility according to the Infectious Diseases Society of America [22, 28]. Jeroense et al. reported performing one‐stage revision arthroplasty in two patients with *Salmonella* prosthetic hip infections, achieving promising outcomes. The authors concluded that, with careful patient selection and appropriate antimicrobial therapy, one‐stage revision can be a successful treatment strategy for managing prosthetic joint infections caused by rare pathogens. However, a DAIR procedure prior to, and after, the one‐stage approach was performed on one of the patients [16]. Osmon et al. recommended that a two‐stage revision arthroplasty is the gold standard for managing chronic prosthetic joint infections, especially those caused by difficult‐to‐treat organisms [28]. Accordingly, as in our case, de la Torre et al. and Tóth et al. point out that a two‐stage revision is optimal to manage *Salmonella* PJI given the virulence of *Salmonella* spp. that can pose significant challenges in case of re‐revision [6, 14].

In the present case, the two‐stage revision approach combined with appropriate antimicrobial therapy resulted in successful eradication of the infection and restoration of joint function. The selection of ceftriaxone, cefepime, and trimethoprim/sulfamethoxazole was guided by their efficacy against *Salmonella enteritidis* and favorable pharmacokinetic profiles, considering the patients′ medical history and previous adverse effects experienced with ciprofloxacin. Extension of the antimicrobial therapy duration with oral transition for several weeks was suggested after consultation from the Department of Infectious Diseases and according to most cases in the literature [4, 5, 10].

## 4. Conclusion

This case underscores the importance of considering rare pathogens like *Salmonella* spp. in the differential diagnosis of late‐onset PJIs, even in patients without typical risk factors or preceding gastrointestinal symptoms. A high index of suspicion, comprehensive diagnostic evaluation, and prompt, aggressive management combining surgical intervention and targeted antimicrobial therapy are crucial for successful outcomes. Currently, there is no definitive evidence favoring one‐stage over two‐stage revision. However, promising outcomes against this pathogen are observed with both options when taking into consideration the guidelines combined with the optimal antibiotic therapy.

## Ethics Statement

Ethical approval was obtained from the Institutional Review Board of KAT General Hospital (IRB No. 1627/2024). Written informed consent was obtained from the patient for publication of this case report and accompanying images.

## Conflicts of Interest

The authors declare no conflicts of interest.

## Funding

All authors have declared that no financial support was received, and they have no financial relationships at present or within the previous years with any organization that might have an interest in the submitted work.

## Data Availability

All relevant deidentified clinical data and images are included within the article. Additional anonymized data are available from the corresponding author upon reasonable request.
